# Immunization by exposure to live virus (SIV_mne_/HIV-2_287_) during antiretroviral drug prophylaxis may reduce risk of subsequent viral challenge

**DOI:** 10.1371/journal.pone.0240495

**Published:** 2021-04-29

**Authors:** Lisa M. Frenkel, LaRene Kuller, Ingrid A. Beck, Che-Chung Tsai, Jaimy P. Joy, Thera M. Mulvania, Shiu-Lok Hu, David C. Montefiori, David M. Anderson

**Affiliations:** 1 Seattle Children’s Research Institute, Seattle, Washington, United States of America; 2 Department of Pediatrics, University of Washington, Seattle, Washington, United States of America; 3 Department of Laboratory Medicine, University of Washington, Seattle, Washington, United States of America; 4 Washington National Primate Research Center (WaNPRC), Seattle, Washington, United States of America; 5 Department of Microbiology, University of Washington, Seattle, Washington, United States of America; 6 Department of Pharmaceutics, University of Washington, Seattle, Washington, United States of America; 7 Duke University Medical Center, Durham, North Carolina, United States of America; CEA, FRANCE

## Abstract

**Rationale/Study design:**

A major challenge in the development of HIV vaccines is finding immunogens that elicit protection against a broad range of viral strains. Immunity to a narrow range of viral strains may protect infants of HIV-infected women or partners discordant for HIV. We hypothesized that immunization to the relevant viral variants could be achieved by exposure to infectious virus during prophylaxis with antiretroviral drugs. To explore this approach in an animal model, macaques were exposed to live virus (SIV_mne_ or HIV-2_287_) during prophylaxis with parenteral tenofovir and humoral and cellular immune responses were quantified. Subsequently, experimental animals were challenged with homologous virus to evaluate protection from infection, and if infection occurred, the course of disease was compared to control animals. Experimental animals uninfected with SIV_mne_ were challenged with heterologous HIV-2_287_ to assess resistance to retroviral infection.

**Methodology/Principal findings:**

Juvenile female *Macaca nemestrina* (N = 8) were given ten weekly intravaginal exposures with either moderately (SIV_mne_) or highly (HIV-2_287_) pathogenic virus during tenofovir prophylaxis. Tenofovir protected all 8 experimental animals from infection, while all untreated control animals became infected. Specific non-neutralizing antibodies were elicited in blood and vaginal secretions of experimental animals, but no ELISPOT responses were detected. Six weeks following the cessation of tenofovir, intravaginal challenge with homologous virus infected 2/4 (50%) of the SIV_mne_-immunized animals and 4/4 (100%) of the HIV-2_287_-immunized animals. The two SIV_mne_-infected and 3 (75%) HIV-2_287_-infected had attenuated disease, suggesting partial protection.

**Conclusions/Significance:**

Repeated exposure to SIV_mne_ or HIV-2_287_, during antiretroviral prophylaxis that blocked infection, induced binding antibodies in the blood and mucosa, but not neutralizing antibodies or specific cellular immune responses. Studies to determine whether antibodies are similarly induced in breastfeeding infants and sexual partners discordant for HIV infection and receiving pre-exposure antiretroviral prophylaxis are warranted, including whether these antibodies appear to confer partial or complete protection from infection.

## Introduction

HIV causes a persistent infection that, without treatment, results in high mortality. Thus, considerable effort has been devoted to the development of a vaccine that can prevent infection (reviewed [1]). Initial efforts to generate a protective HIV vaccine were largely focused on eliciting protective immunity via broadly neutralizing antibodies (reviewed [2]). This approach was pursued due to observations that sera of chronically HIV-infected individuals neutralized significant numbers of heterologous virus isolates [3], and the transfer of sera containing neutralizing HIV/SIV antibodies and neutralizing monoclonal antibodies have protected experimental animals from mucosal challenge [4–6]. However, the breadth of vaccine-elicited neutralizing antibodies has generally been narrow (reviewed [7]). Also problematic is that in most individuals, the induction of broadly neutralizing antibodies requires a series of mutations in V-beta chains that occurs over months to years [8].

Many individuals at-risk of HIV infection, particularly breastfeeding infants of infected mothers and sexual partners discordant for HIV infection, are repeatedly exposed to the same viral “swarm” or quasispecies from mother’s breast milk or his/her infected partner’s genital fluids. These individuals could potentially benefit from a “narrow” immune response to the HIV swarm during repeat viral exposures. We hypothesized that a specific immune response could be induced to the relevant virus and protect the susceptible individual from HIV infection. In support of this are the observations that: (1) Seronegative sexual partners of HIV-infected individuals with undetectable plasma viral loads develop HIV-specific CD8+ T cell responses, suggesting that low, but persistent, exposure to HIV may be sufficient to elicit virus-specific immune responses [9,10] and (2) The combination of an Env vaccine and pre-exposure prophylaxis (**PrEP**), tenofovir microbicide gel, reduced the risk of HIV infection more than either intervention alone [11]. Taken together, repeated viral exposures during PrEP with antiretrovirals may induce protective immune responses to a specific viral quasispecies. PrEP with antiretrovirals is recommended for individuals at risk of HIV infection in the US and globally based on several clinical trials (CDC & WHO, 2015) [12–15]. Individuals on PrEP can develop mucosal HIV-1-specific IgA immune responses [16], which may protect them from HIV acquisition.

The current study explores in a controlled trial of macaques whether multiple exposures to infectious virus during antiretroviral prophylaxis induces specific immunity to the relevant viruses by repeated exposures during antiretroviral prophylaxis. Each animal’s binding and neutralizing antibodies and cellular immune responses were measured. Subsequently, the macaques were challenged with homologous virus. If uninfected after three homologous viral challenges, then a heterologous viral challenge was administered to determine if the animal was susceptible to retroviral infection. Infected animals were monitored to evaluate whether the disease course was attenuated compared to controls.

## Methods

### Study animals and design

The animals used for study were pre-pubertal female *Macaca nemestrina*, clinically healthy by physical examination, serum chemistries, and complete blood counts, including negative serology and polymerase chain reaction assay (**PCR**) of peripheral blood mononuclear cells (**PBMC**) for simian retrovirus (**SRV**) infection. The macaques were housed singly in stainless steel cages in the Animal Biosafety Level 2-Plus facilities at the accredited Washington National Primate Research Center (**WaNPRC**). Animal care and husbandry met or exceeded the standards described in “The Guide for the Care and Use of Laboratory Animals” published by the Institute of Laboratory Animal Resources Commission on Life Sciences, National Research Council. All procedures were approved by the University of Washington’s Institutional Animal Care and Use Committee.

**Fig 1:**
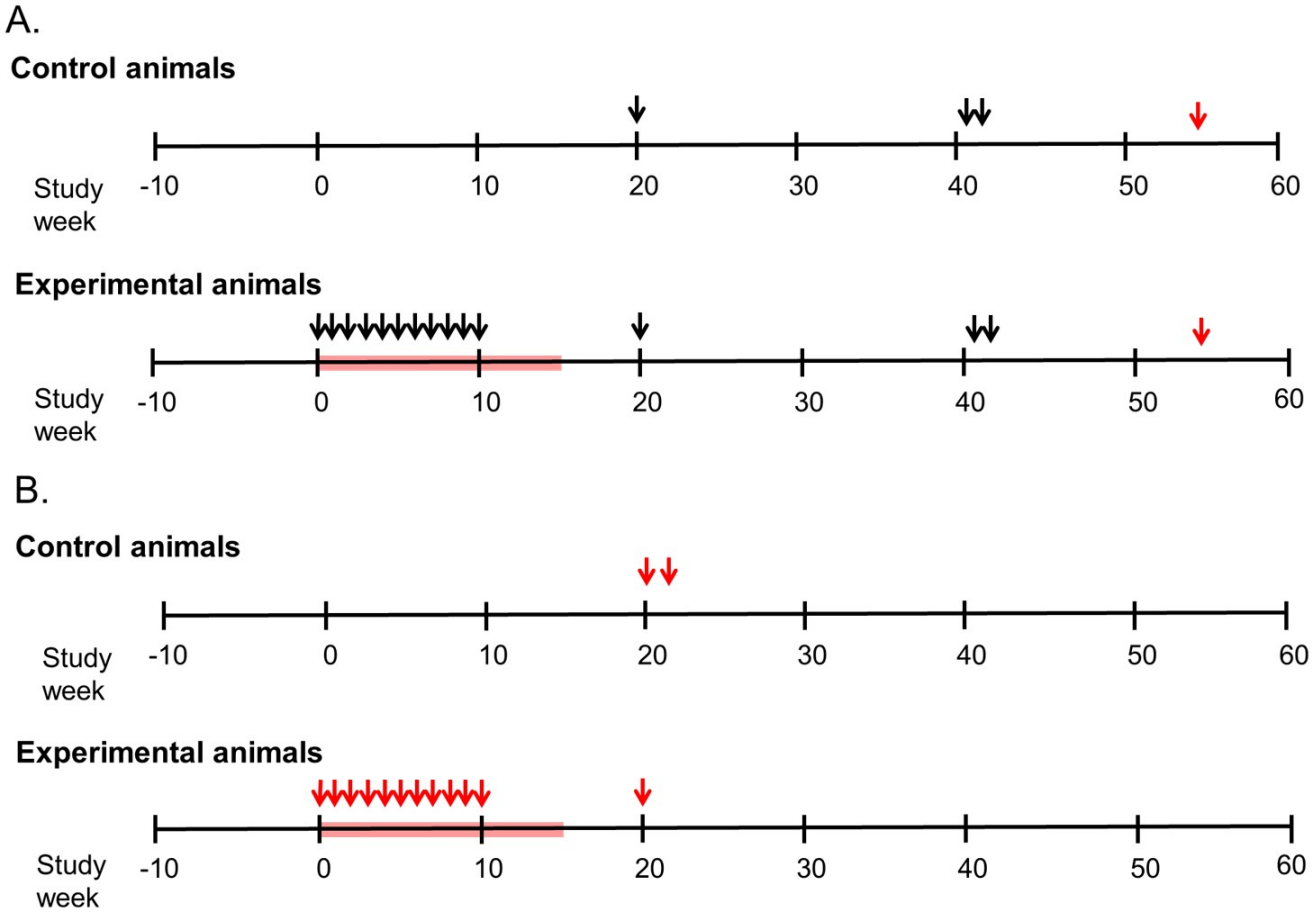
Study design. **A**. Time schema for immunization of experimental group (n = 4) of female juvenile *Macaca nemestrina* given ten weekly intravaginal exposures to SIV_mne_ at a TCID_50_ of 10^4^ (arrows on left) during tenofovir (red block). Controls did not receive tenofovir and were similarly inoculated (n = 2 animals) or given two inoculations 24 hours apart (n = 2 animals). Macaques were intravaginally challenged initially (at Study Week 20) with 10^4^ TCID_50_ of SIV_mne_ grown in homologous cells (estimated 10 x the 100% animal infectious dose). Uninfected animals were subsequently challenged (at Study Week 41) with SIV_mne_ (two doses of 10^4.5^ TCID_50_ grown in allogenic animals and given 24 hours apart). To determine if uninfected animals were susceptible to lentivirus infection, they were inoculated with HIV-2_287_ (at Study Week 54). Blood and vaginal secretions were collected weekly over ten weeks, then every 2–6 weeks, and inguinal lymph nodes collected at Study Week 19. Specimens were assessed for viral RNA, DNA, infectivity, binding and neutralizing antibodies and cellular responses to Gag. **B**. Panel B shows the time schema for immunization and challenges of HIV-2_287_ experimental group (n = 4). The study designed mirrored that in Panel A, except that the control animals (n = 4) were only given two intravaginal exposures, and the experimental animals were only given one intravaginal challenge (at Study Week 20).

The study design (Fig 1) included administration of tenofovir, ({[(2*R*)-1-(6-amino-9*H*-purin-9-yl)propan-2-yl]oxy}methyl)phosphonic acid, 20 mg/kg by subcutaneous injection to experimental macaques once daily. The control animals were injected with normal saline as a placebo. The experimental and control macaques were inoculated intra-vaginally once a week for 10 weeks with 10^4^ tissue culture infectious dose for 50% of wells (**TCID**_**50**_) of virus, which was estimated to represent ten 100% animal infectious doses grown in autologous PBMC. The virus was introduced through a soft plastic 2.5 Fr nasal/gastric feeding tube into the vaginal vault followed by a flush of 1 mL sterile PBS.

### Generation of viral stocks

Virus stocks were generated for each animal using autologous PBMCs to avoid viral clearance on the basis of macaque allo-antigens incorporated into the viral envelope [17,18].

Phytohemoglutinin-A (**PHA**) stimulated CD8-depleted PBMCs from each macaque were infected with SIV_mne_ working stock B2455-1b or HIV-2_287_ working-stock-8 [19]. The cultures were monitored for cytopathic effects, specifically syncitia formation, and p27 antigen (**Ag**) production. At the peak of syncitia and p27Ag expression, uninfected CD8-depleted PBMCs from each macaque were independently added to the virus culture to allow a second phase of viral replication. The supernatant was subsequently filtered with a Centricon Plus-80 with Biomax-PB membrane (NMWL100,000) (Millipore, Bedford, MA) and stored in liquid nitrogen to be used as viral stock for each macaque made from her own PBMCs. To estimate the infectious titer of the stock viruses, a ten-fold dilution series using one stock aliquot each were carried out using 10^6^ macaque CD8-depleted PBMCs in quadruplicate across a 48-well plate. After maintenance of the cultures for 10 days, the viral antigen in the supernatant was measured and the TCID_50_/mL of the stock calculated.

### SIV/HIV-2 cultures of PBMCs with and without depletion of CD8^+^ T cells

The human CD4 T/B hybrid cell line, CEMx174, was used for SIV isolation [20], and CD8-depleted human PBMCs for HIV-2 isolation [21]. Two successive increases in antigen supernatant levels were used to define a positive culture. The number of HIV-2 positive cells was determined and reported as infectious units per million cells (**IUPM**).

### Quantitative PCR for SIV and HIV-2 plasma RNA

Plasma viral SIV RNA levels were determined using quantitative PCR (Bayer, Emeryville, CA) with a lower limit of detection of 1,000 copies/mL of plasma [22]. HIV-2 RNA plasma levels were quantified as described [21]. The lower limit of quantification of the HIV-2 assay was 1580 copies/mL.

### SIV/HIV-2 DNA PCR

Total DNA was extracted from isolated PBMC or tissues after grinding with mortar and pestle using the IsoQuick Nucleic Acid Extraction Kit (Orca Research, Bothell, WA). Nested PCR was performed on 2 ug of DNA (equivalent to ~300,000 cells) per reaction utilizing primers specific for the LTR-gag region of SIV_mne_ and HIV-2_287_. The primers used were G1/G2B and S1/G4 (**G1**: TCTCTCCTAGTCGCCGCCTGGT; **G2B**: TTCATTTGCTGCCCATACTACA; **S1**: CGATAATAAGAAGACCCTGGTCTG and **G4**: TTCTTCCCTGACAAGACGGAG). The first-round cycling conditions were: 40 cycles of 94°C for 30 sec, 57°C for 30 sec and 72°C for 1 min, followed by an extension step of 72°C for 7 minutes. Two uL of first round product was used in the second round, the second-round cycling conditions were: 35 cycles of 94°C for 30 sec, 57°C for 30 sec and 72°C for 1 min, followed by an extension step of 72°C for 7 minutes. The 320-bp DNA fragment amplified was visualized by electrophoresis on 2% agarose gels and ethidium bromide staining. The limit of detection was 3–10 copies/10^6^ cells.

### Differentiation between infection by SIV_mne_ or HIV-2_287_

The viral nucleic acids detected by PCR of the LTR-*gag* region were digested with the restriction endonuclease ScaI (New England BioLabs, Beverly, MA), which cuts within the amplicon of HIV-2_287_ but not SIV_mne_. Five uL of each PCR product were digested with ScaI for 6 hours at 37°C. The digestion products, two DNA fragments of 240 and 80-bp for HIV-2 or one fragment of 320-bp for SIV, were separated on a 3% agarose gel and stained with ethidium bromide.

### Lymphocyte subset determinations in macaques

Absolute counts and ratios of CD4+ and CD8+ cells in the peripheral blood of virus-infected macaques were determined by standard fluorescent activated cell sorting (**FACS**) procedures using antibodies previously shown to react with macaque cells [20,23].

### IFN-γ ELISpot to Gag

Functional CD8 T-cell activity was measured and quantified using a cytokine ELISpot assay [24,25]. MHC Class I antigen presenting cells were constructed by infection of autologous *H*. *papio* transformed B cells with recombinant vaccinia viruses expressing *gag* of SIV, and combined with PBMCs at an effector to target ratio (**E:T**) of 2:1. Following an 18 hour incubation at 37°C, the cells were transferred in duplicate 2-fold dilutions from 2x10^5^-2.5x10^4^ cells/mL into an ELISpot plate that was pre-coated with monoclonal antibodies directed against macaque IFN-γ, and the assay done following the manufacturer’s instructions (U-Cytech, Utrecht, the Netherlands). The IFN-γ captured on the plate was detected by labeling with biotinylated polyclonal anti-IFN-γ antibodies followed by ϕ-labeled anti-biotin antibodies, and a proprietary activator that allows “spot” formation. The spots were counted using an ELISpot plate reader (Zeiss, Thornwood, New York). The average number of spots per well was compared to the number of input T cells, and linear regression used to determine the strength of the relationship made by the four data points (R^2^ value). The slope of the line indicated the frequency of IFN-γ secreting cells. The "partial F test" was utilized to determine if the experimental line was different from the control line.

### Quantitative total IgG antibody ELISA

Total antibody isotypes were determined using published methods [26]. A standard curve consisting of known amounts of purified IgG (Sigma, St. Louis MO) was run in conjunction with each assay. IgG levels in the samples were quantitatively interpolated from the standard curve by applying the linear regression equation obtained from the standard curve to the optical density values of the experimental samples. The reproducibility of the quantitative isotype-specific ELISA assay was evaluated by comparing the linear portion of both the experimental and standard sample curves (n = 38). The standard deviation (**SD**) ranged from 1.3 x 10^−6^ to 3.6 x 10^−8^ between the observed antibody concentrations (experimental curves) and the known antibody concentrations (standard curves). A coefficient of variance (**CV**) of 1% to 5% indicated that the assay performed consistently.

### Quantitative SIV_mne_ and HIV-2_287_ antibody

Determination of virus-specific antibody levels was performed as previously described [27]. Virus-specific IgG levels were derived from the standard curve (run concurrently) as described above for the total IgG ELISA. The lowest level of detection for the quantitative ELISA is 1 x 10^−5^ μg antibody/mL. Reproducibility of the ELISA was evaluated by measuring the mid-point optical density of the linear portion of the standard curve for each assay (n = 20). The SD for the SIV_mne_-specific ELISA was 0.072 and 0.066 for the HIV-2_287_ ELISA. A CV of 8.2% indicated that the assays performed consistently.

### Neutralizing antibodies to SIV_mne_ or HIV-2_287_

Neutralizing antibodies against SIV_mne_ or HIV-2_287_ were assessed in CEMx174 cells as described previously [28]. Briefly 50 μL of cell-free virus (5,000 TCID_50_) was added to multiple dilutions of test serum in 100 μL of growth medium in triplicate wells of 96-well microtiter plates and incubated for 1 hr at 37°C. Cells (7.5 x 10^4^) in 100 μL of growth medium were added and incubated until extensive syncytium formation and nearly complete cell-killing were evident microscopically in virus control wells. Cell densities were reduced and medium replaced after 3 days incubation when more than 3 days were required to reach the assay end-point. Viable cells were stained with Finter’s neutral red in poly-L-lysine coated plates. Percent protection from virus-induced cell-killing was determined by calculating the difference in absorption (A_540_) between test wells (cells + serum sample + virus) and virus control wells (cells + virus), dividing this result by the difference in absorption between cell control wells (cells only) and virus control wells and multiplying by 100. Neutralizing antibody titers are expressed as the reciprocal of the serum dilution that protected 50% of cells from virus-induced killing. This cut-off corresponds to an approximate 90% reduction in p24 antigen synthesis. Assay stocks of virus were produced in either H9 cells (SIV_mne_) or human PBMC (HIV-2_287_).

### Statistical analysis

The viral infectivity and CD4+ T cell concentrations between control and experimental animals were determined using a mixed-effects model with Geisser-Greenhouse’s correction.

## Results

### Tenofovir prophylaxis protected animals from infection

**Fig 2:**
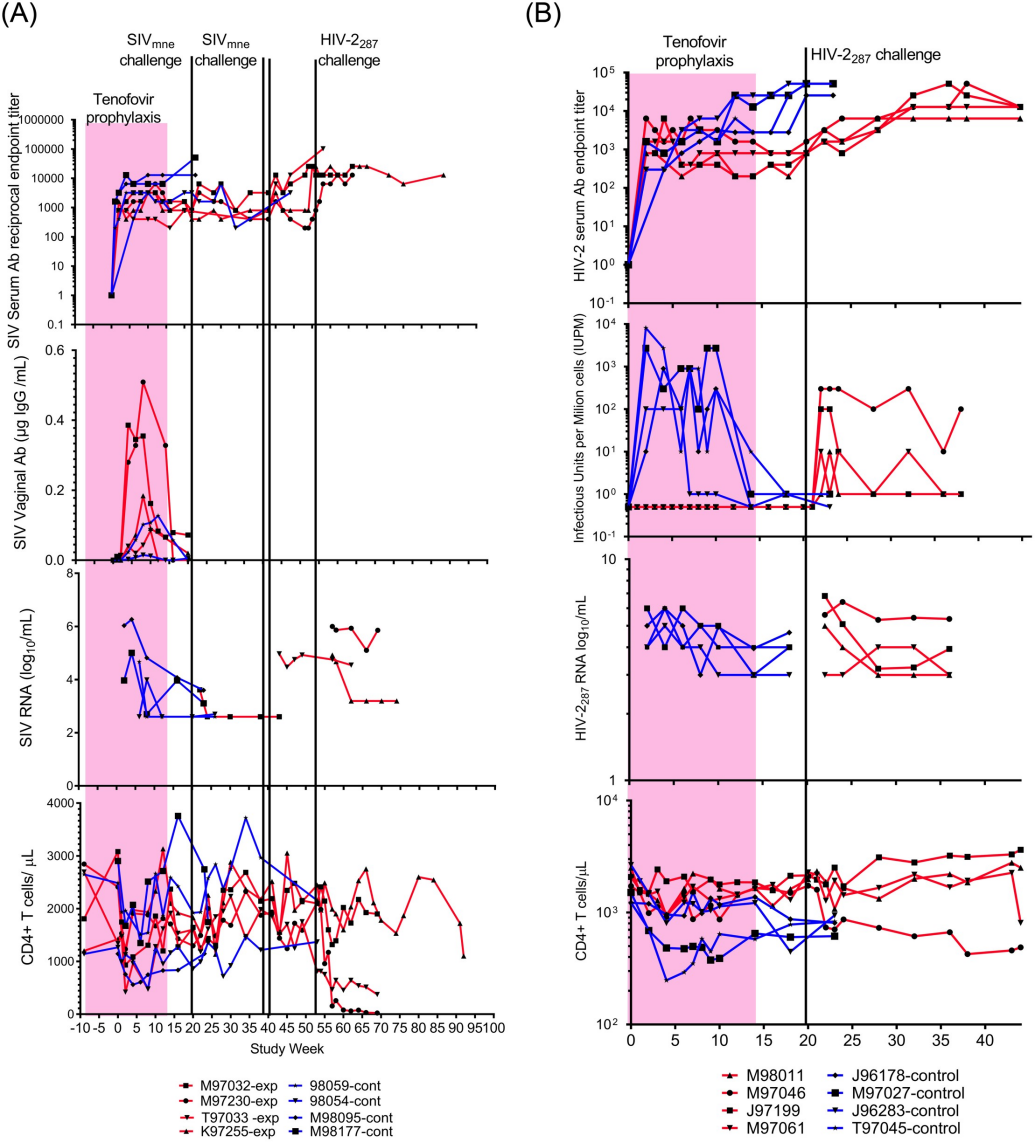
Macaques immunized to SIV/HIV-2 by exposure to virus during tenofovir prophylaxis resisted viral challenge and demonstrated protection from disease. **A**. Panels show SIV serum (top) and vaginal (second from top) IgG antibody levels, plasma viral load (third from top), and peripheral blood CD4^+^ lymphocyte concentrations (bottom) for control (blue lines) and experimental (red lines) macaques. Tenofovir (pink block) was given to experimental group from Study Week 0–14. Experimental animals had intravaginal challenges with SIV_Mne_ (green lines). One experimental animal, M97032, was infected by the first challenge of 10^4^ TCID_50_ of SIV_mne_ grown in homologous cells. A second animal, T97033, was infected by the second/third challenge with two doses of 10^4.5^ TCID_50_ SIV_mne_ grown in heterologous cells separated by 24 hours. Two control animals (M98095 and M98177) similarly received two intravaginal inoculations from this viral pool. The remaining two experimental animals, M97230 and K97255, were not infected by SIV_mne_ challenges; but were not resistant to retroviruses as infected by HIV-2_287_ challenge (black line). Note, vaginal antibody is higher after immunizing exposures in experimental four animals compared to two controls evaluated, likely due to greater number of intravaginal SIV exposures (10 vs. 2) in these animals. **B**. Panels show HIV-2_287_ serum IgG antibody levels (top), viral load by co-culture (second from top), plasma HIV-2 RNA load (third from top; limit of detection above gray block), and CD4^+^ lymphocyte concentrations (bottom) for control (blue lines) and experimental (red lines) macaques; tenofovir administration (pink block), and one HIV-2_287_ challenge (black line). Animals “immunized” with HIV-2_287_ demonstrated protection from disease (not infection) on challenge with homologous virus, with significantly lower viral infectivity (P = 0.05) and maintenance of CD4^+^ T lymphocyte concentrations compared to controls (P = 0.01). Significance was calculated using a mixed-effects analysis with Geiser-Greenhouse correction.

Antiretroviral prophylaxis with tenofovir given immediately prior to or at 24 hours following each of ten weekly intravaginal exposures to HIV-2_287_ or SIV_mne_ protected all 8 macaques from infection. In comparison, all placebo-treated controls became infected (Fig 2). Weekly monitoring for viral DNA were negative for all the experimental macaques’ PBMC at study weeks 1–14 during tenofovir administration (multiple PBMC aliquots totaled 14–30 μg DNA/animal). After cessation of tenofovir, multiple PBMC specimens were negative for virus by co-culture and PCR (6–10μg DNA/animal collected over study weeks 15 to 20) as was inguinal lymphoid tissue collected at study week 19 (total of 20–30 μg DNA/animal, each PCR with 2 μg DNA).

Control animals not administered tenofovir prophylaxis all became infected by the intravaginal inoculations. Weekly blood sampling initially detected viral DNA by PCR at Study Week 4, 5, 2 and 2 in the four the SIV_mne_ control animals and at Study Week 2 in all four HIV-2_287_ control animals. Once infected, virus was consistently detected throughout the study in the control animals by co-culture and RNA and DNA PCR. CD4 cells decreased rapidly in the HIV-2_287_ infected control animals (Fig 2B).

### Induction of antibodies by exposure to virus during tenofovir prophylaxis

Exposure of the experimental animals to virus during tenofovir prophylaxis induced specific, non-neutralizing antibody in sera to SIV_mne_ or HIV-2_287_. The antibody concentrations in uninfected experimental animals were similar to the infected control animals (Fig 2). Vaginal antibodies to SIV were compared between the tenofovir-treated and control animals during study weeks 1–20, and the tenofovir-treated animals appeared to achieve higher levels (Fig 2A).

Neutralizing antibodies were not induced by the vaginal exposures during tenofovir prophylaxis assessed in sera at Study Week 20 just prior to viral challenge. However, neutralizing antibodies were detected in infected control animals, and in all experimental macaques once infected by a challenge virus (Table 1).

**Table 1:** Infected control animals and virus challenged experimental macaques demonstrated detectable neutralizing antibodies and cellular immune responses. **A**. NAb titers to SIVmne and Gag ELISpot results for control an experimental macaques at study weeks 0,16, and/or 38. **B**. NAb titers to HIV2-287 and Gag ELISpot results for control an experimental macaques at study weeks 0, 10, and 20.

Group	Animal ID	Bleed Wk	NAb titer* to SIVmne	Gag ELISpot	Animal ID	Bleed Week	NAb titer* to HIV2-287	Gag ELISpot
**Control**	98054	0	<30	-	M97027	0	96	-
		16	30	+		10	73	nt
	98059	0	<30	-		20	95	+
		16	75	-	T97045	0	<20	-
	M98095	0	<30	nt		10	<20	nt
		16	1,990	nt		20	192	+
	M98177	0	<30	nt	J96178	0	<20	-
		16	326	nt		10	<20	nt
**Experimental**	M97032	0	<30	-		20	68	+
		16	<30	-	J96283	0	<20	+/-
	T97033	0	<30	-		10	<20	nt
		16	<30	-		20	<20	++/+
		38	<30	nt	M98011	0	46	+
	M97230	0	<30	-		10	157	nt
		16	<30	-		20	179	++
		38	<30	nt	M97046	0	214	+
	K97255	16	<30	-		10	89	nt
		38	<30	nt		20	47	+

*NAb titers are reported as reciprocal plasma dilution at which 50% of cells (CEMx174) were protected from virus induced killing. ELISpot reactivity to viral antigen vs. medium was graded as (-) = no increase; (+) = increased; (++) = markedly increased; (nt) = not tested.

### Cellular immune responses after exposure to virus during tenofovir prophylaxis

SIV/HIV-specific cellular responses (IFN-γ ELISpot to Gag) were not induced in the experimental animals by viral exposures during tenofovir prophylaxis. ELISpot responses were detected in control animals following infection, and in experimental animals after infection with challenge viruses (Table 1).

### Viral challenge of SIV_mne_-immunized macaques

At Study Week 20, (six weeks after tenofovir was stopped, ten weeks after the final intravaginal inoculation during chemoprophylaxis), the SIV_mne_-immunized animals were challenged with 10^4^ TCID_50_ of SIV_mne_ (Fig 2A). Importantly, the SIV_mne_ used for intravaginal immunization and through the first challenge were grown in each animal’s autologous cells to avoid clearance based on allo-antigens incorporated into the viral envelope. Following this first challenge, one (M97032) of the four tenofovir-treated animals became infected. SIV DNA was persistently detected in the PBMC from this macaque beginning at Study Week 22 (two weeks post-intravaginal challenge), although viral co-cultures of this animal’s PBMC were rarely positive. After the initial challenge, SIV_mne_ was not detected in the other three macaques’ PBMC; neither by DNA PCR (6 μg DNA across 4 specimens/animal) nor by weekly co-cultures (6 cultures/animal).

The three uninfected experimental animals were challenged a second and third time with 10^4.5^ TCID_50_ SIV_mne_ grown in allogenic animals at Study Week 41 (21 weeks after the first challenge, 31 weeks after the last immunizing exposures during tenofovir prophylaxis). Two inoculations, 24 hours apart, with a greater quantity of virus, were administered due to the observation that the first two SIV_mne_ control macaques given weekly intravaginal inoculations of 10^4^ TCID_50_ did not have virus detected in their PBMC within the expected time frame. Virus is typically detected one to three weeks following intravaginal inoculation of macaques with 10^4^ TCID_50_ SIV_mne_, (unpublished, WaNPRC). One control animal had virus first detected by DNA PCR and culture five weeks following initial inoculation, the other had virus detected after four weeks by PCR and after five weeks by culture. This delay in the detection of virus suggested decreased infectivity of the inoculum. Several explanations were considered. First, while 10^4^ TCID_50_ was 10 times the 100% animal infectious dose in previous titration experiments, these data were limited to two animals per dose level (WaNPRC, unpublished), and infection by the intravaginal route is known to be relatively inconsistent [29]. Second, the virus could have been attenuated by the additional passage in the macaques’ own PBMC. Finally, the absence of disparate MHC antigens on the viral envelope could reduce infectivity as it is likely that fewer intravaginal lymphocytes became activated. Therefore, a greater amount of SIV_mne_ (two doses of 10^4.5^ TCID_50_ given 24 hours apart), and with virus grown in allogenic animals, was used for the second and third challenge of the three uninfected experimental animals. Two additional control animals were similarly challenged with the pool of virus grown in allogenic animals, with SIV_mne_ infection detected two weeks following the inoculation, these control animals had detectable viremia by both culture and DNA PCR.

After the second and third challenge in the experimental group, one (T97033) of the three macaques became infected (Fig 2A). SIV was detected in co-culture and by DNA PCR two weeks after the challenge and persistently thereafter. The two uninfected macaques each had seven negative PBMC cultures and DNA PCR (total of 7 μg PBMC/animal) from blood collected every other week over a period of 13 weeks. These two animals were subsequently challenged and infected with HIV-2_287_ (Fig 2A). The viral loads and CD4 cell counts differed between these two animals, one (K97255) had attenuated disease, with low levels of plasma HIV-2 RNA and normal CD4 counts.

### Viral challenge of HIV-2_287_-immunized macaques

At Study Week 20, ten weeks following the last immunizing HIV-2_287_ intravaginal inoculation (six weeks following the final dose of tenofovir), the experimental macaques were challenged with HIV-2_287_ grown in autologous PBMCs. All four immunized animals became infected. While HIV-2_287_ RNA loads appeared similar, disease was attenuated in three of the four animals compared to controls, with lower HIV-2_287_ loads in quantitative co-culture (P = 0.05), and delayed loss of CD4 cells (P = 0.01) (Fig 2B).

## Discussion

Our strategy to immunize macaques to a specific strain of virus by exposure to live SIV_mne_ or HIV-2_287_ during protective antiretroviral prophylaxis with tenofovir resulted in the generation of specific antibodies by all animals. Half of the SIV_mne_ immunized animals appeared to resist challenge with homologous virus. While the HIV-2_287_ immunized animals did not appear to resist infection, their disease was attenuated with lower concentrations of infectious virus and maintenance of normal CD4 cells counts. This macaque model suggests that partial protection to HIV could be achieved by exposure to infectious virus during antiretroviral prophylaxis.

The mechanism by which our immunization strategy may have blocked virus transmission to some animals and limited viral replication in others is uncertain. Tenofovir prophylaxis would have allowed virus to enter the animals’ cells but blocked reverse transcription of viral RNA necessary to establish infection. Virus-specific immune responses induced during tenofovir prophylaxis included binding antibodies in both the serum and vaginal secretions, but no detectable neutralizing antibodies or virus specific cellular responses. We speculate that when the macaques were later challenged with live homologous virus, the binding antibodies may have contributed to protection in the SIV_mne_-exposed animals and to attenuation of disease in the HIV-2_287_ group. The virus specific IgG may have partially protected macaques from infection by antibody dependent cellular toxicity (**ADCC**) [30]. Vaginal secretory IgA has correlated with protection of high-risk humans from HIV infection [31,32], and macaques [33], which supports our contention that vaginal IgA may have blocked infection by binding virus. However, serum IgA was shown to antagonize the effects of IgG-mediated ADCC in other studies [34,35]; and vaginal secretory IgA has not been observed in all exposed-uninfected individuals [16,36–38].

In the animals with attenuated disease, antibodies may have had diminished viral infectivity, as antibodies appear to modulate the level of viremia after the first month of infection [39]. In fact, passive transfer of neutralizing IgG in 1-month-old HIV-infected rhesus macaques resulted in reduced plasma and PBMC-associated viremia [40]. Alternatively, allelic differentiation in viral antigen presentation due to genetic diversity among rhesus macaques may have contributed to attenuation of disease [41] (reviewed in [42]).

While assays using PBMC did not reveal cellular immune responses, it is conceivable that dendritic cells in the vaginal mucosa initiated specific cellular immune responses limited to the mucosa or regional nodes [43]. The nodes draining the vagina of our experimental animals were not examined for viral nucleic acids, proteins or cellular responses, as these are difficult to access surgically, including at necropsy. One limitation of our study is the small sample size, which precluded highly statistically significant findings. In addition, non-human primate models using SIV and HIV-2 do not accurately mirror human infection with HIV. Atraumatic introduction of virus into the vagina, used in this model system, differs from sexual intercourse where trauma [43] or ulcerations may perturb mucosal barriers [44], which could overcome binding antibody. The animals we studied were healthy, while humans frequently have sexually transmitted infections that could increase lymphocytes that serve as viral targets in the mucosa surface [45]. The safety of our immunization strategy is uncertain as pre-exposure prophylaxis has been noted to fail [46]. Vaginal immunization could increase the concentration of target cells in the vaginal mucosa, which has been speculated to increase susceptibility to HIV infection [45]. However, it is important to note that the production of systemic antibody and attenuated disease in infected macaques indicates that the mucosal exposures did not induce tolerance to viral antigens. Similar to induction of antibody responses by intravaginal immunizations in the animals we studied, other studies have detected humoral and cellular immune responses after intravaginal [47,48], rectal [49,50] and nasal [51–54] administration of HIV/SIV. These observations suggest that administering prophylaxis during HIV exposures may effectively immunize individuals discordant for HIV infection, such as monogamous sexual partners and/or breastfeeding infants.

In summary, we explored a novel strategy to immunize macaques to homologous lentiviruses by exposure to live virus during PrEP. This model is relevant to monogamous sexual partners discordant for HIV infection and breastfeeding infants, as both could benefit from protective immunity to homologous virus. Our experiments found this strategy induced specific binding antibodies and suggested that after discontinuation of PrEP, some macaques resisted challenge with homologous virus. However, additional studies are needed to further test this strategy and define the mechanisms and increase resistance to challenges with infectious virus.

## References

[1] Gao Y, McKay PF, Mann JFS. Advances in HIV-1 Vaccine Development. Viruses. 2018;10(4). 10.3390/v10040167 29614779 PMC5923461

[2] Pegu A, Hessell AJ, Mascola JR, Haigwood NL. Use of broadly neutralizing antibodies for HIV-1 prevention. Immunol Rev. 2017;275(1):296–312. 10.1111/imr.12511 28133803 PMC5314445

[3] Deeks SG, Schweighardt B, Wrin T, Galovich J, Hoh R, Sinclair E, et al. Neutralizing antibody responses against autologous and heterologous viruses in acute versus chronic human immunodeficiency virus (HIV) infection: evidence for a constraint on the ability of HIV to completely evade neutralizing antibody responses. J Virol. 2006;80(12):6155–64. 10.1128/JVI.00093-06 16731954 PMC1472617

[4] Baba TW, Liska V, Hofmann-Lehmann R, Vlasak J, Xu W, Ayehunie S, et al. Human neutralizing monoclonal antibodies of the IgG1 subtype protect against mucosal simian-human immunodeficiency virus infection. Nat Med. 2000;6(2):200–6. 10.1038/72309 10655110

[5] Mascola JR, Stiegler G, VanCott TC, Katinger H, Carpenter CB, Hanson CE, et al. Protection of macaques against vaginal transmission of a pathogenic HIV-1/SIV chimeric virus by passive infusion of neutralizing antibodies. Nat Med. 2000;6(2):207–10. 10.1038/72318 10655111

[6] Veazey RS, Shattock RJ, Pope M, Kirijan JC, Jones J, Hu Q, et al. Prevention of virus transmission to macaque monkeys by a vaginally applied monoclonal antibody to HIV-1 gp120. Nat Med. 2003;9(3):343–6. 10.1038/nm833 12579198

[7] Montefiori DC, Roederer M, Morris L, Seaman MS. Neutralization tiers of HIV-1. Curr Opin HIV AIDS. 2018;13(2):128–36. 10.1097/COH.0000000000000442 29266013 PMC5802254

[8] Klein F, Diskin R, Scheid JF, Gaebler C, Mouquet H, Georgiev IS, et al. Somatic mutations of the immunoglobulin framework are generally required for broad and potent HIV-1 neutralization. Cell. 2013;153(1):126–38. 10.1016/j.cell.2013.03.018 23540694 PMC3792590

[9] Restrepo C, Rallon NI, del Romero J, Rodriguez C, Hernando V, Lopez M, et al. Low-level exposure to HIV induces virus-specific T cell responses and immune activation in exposed HIV-seronegative individuals. J Immunol. 2010;185(2):982–9. 10.4049/jimmunol.1000221 20543099

[10] Makedonas G, Bruneau J, Lin H, Sekaly RP, Lamothe F, Bernard NF. HIV-specific CD8 T-cell activity in uninfected injection drug users is associated with maintenance of seronegativity. AIDS. 2002;16(12):1595–602. 10.1097/00002030-200208160-00004 12172080

[11] Le Grand R, Dereuddre-Bosquet N, Dispinseri S, Gosse L, Desjardins D, Shen X, et al. Superior Efficacy of a Human Immunodeficiency Virus Vaccine Combined with Antiretroviral Prevention in Simian-Human Immunodeficiency Virus-Challenged Nonhuman Primates. J Virol. 2016;90(11):5315–28. 10.1128/JVI.00230-16 27009957 PMC4934744

[12] Baeten JM, Donnell D, Ndase P, Mugo NR, Campbell JD, Wangisi J, et al. Antiretroviral prophylaxis for HIV prevention in heterosexual men and women. N Engl J Med. 2012;367(5):399–410. 10.1056/NEJMoa1108524 22784037 PMC3770474

[13] Choopanya K, Martin M, Suntharasamai P, Sangkum U, Mock PA, Leethochawalit M, et al. Antiretroviral prophylaxis for HIV infection in injecting drug users in Bangkok, Thailand (the Bangkok Tenofovir Study): a randomised, double-blind, placebo-controlled phase 3 trial. Lancet. 2013;381(9883):2083–90. 10.1016/S0140-6736(13)61127-7 23769234

[14] Grant RM. Antiretroviral agents used by HIV-uninfected persons for prevention: pre- and postexposure prophylaxis. Clin Infect Dis. 2010;50 Suppl 3:S96–101. 10.1086/651479 20397962 PMC3663282

[15] Thigpen MC, Kebaabetswe PM, Paxton LA, Smith DK, Rose CE, Segolodi TM, et al. Antiretroviral preexposure prophylaxis for heterosexual HIV transmission in Botswana. N Engl J Med. 2012;367(5):423–34. 10.1056/NEJMoa1110711 22784038

[16] Lund JM, Broliden K, Pyra MN, Thomas KK, Donnell D, Irungu E, et al. HIV-1-Neutralizing IgA Detected in Genital Secretions of Highly HIV-1-Exposed Seronegative Women on Oral Preexposure Prophylaxis. J Virol. 2016;90(21):9855–61. 10.1128/JVI.01482-16 27558421 PMC5068535

[17] Murphey-Corb M, Martin LN, Davison-Fairburn B, Montelaro RC, Miller M, West M, et al. A formalin-inactivated whole SIV vaccine confers protection in macaques. Science. 1989;246(4935):1293–7. 10.1126/science.2555923 2555923

[18] Stott EJ. Anti-cell antibody in macaques. Nature. 1991;353(6343):393. 10.1038/353393a0 1815549

[19] Pettifor AE, Hudgens MG, Levandowski BA, Rees HV, Cohen MS. Highly efficient HIV transmission to young women in South Africa. AIDS. 2007;21(7):861–5. 10.1097/QAD.0b013e3280f00fb3 17415041

[20] Morton WR, Kuller L, Benveniste RE, Clark EA, Tsai CC, Gale MJ, et al. Transmission of the simian immunodeficiency virus SIVmne in macaques and baboons. J Med Primatol. 1989;18(3–4):237–45. 2547959

[21] Watson A, McClure J, Ranchalis J, Scheibel M, Schmidt A, Kennedy B, et al. Early postinfection antiviral treatment reduces viral load and prevents CD4+ cell decline in HIV type 2-infected macaques. AIDS Res Hum Retroviruses. 1997;13(16):1375–81. 10.1089/aid.1997.13.1375 9359657

[22] Leutenegger CM, Higgins J, Matthews TB, Tarantal AF, Luciw PA, Pedersen NC, et al. Real-time TaqMan PCR as a specific and more sensitive alternative to the branched-chain DNA assay for quantitation of simian immunodeficiency virus RNA. AIDS Res Hum Retroviruses. 2001;17(3):243–51. 10.1089/088922201750063160 11177407

[23] Firpo PP, Axberg I, Scheibel M, Clark EA. Macaque CD4+ T-cell subsets: influence of activation on infection by simian immunodeficiency viruses (SIV). AIDS Res Hum Retroviruses. 1992;8(3):357–66. 10.1089/aid.1992.8.357 1349228

[24] Mulvania T, Lynch JB, Robertson MN, Greenberg PD, Morton WR, Mullins JI. Antigen-specific cytokine responses in vaccinated Macaca nemestrina. J Med Primatol. 1999;28(4–5):181–9. 10.1111/j.1600-0684.1999.tb00268.x 10593484

[25] Mulvania T, Coon E, Kuller L, Agy MB, Morton WR, Mullins JI. Natural history of SIVmac BK28 and H824 infection in Macaca nemestrina. J Med Primatol. 1998;27(2–3):87–93. 10.1111/j.1600-0684.1998.tb00231.x 9747948

[26] Kuller L, Morton WR, Benveniste RE, Tsai CC, Clark EA, Gale MJ, et al. Inoculation of Macaca fascicularis with simian immunodeficiency virus, SIVmne immunologic, serologic, and pathologic changes. J Med Primatol. 1990;19(3–4):367–80. 2231689

[27] Hu SL, Zarling JM, Chinn J, Travis BM, Moran PA, Sias J, et al. Protection of macaques against simian AIDS by immunization with a recombinant vaccinia virus expressing the envelope glycoproteins of simian type D retrovirus. Proc Natl Acad Sci U S A. 1989;86(18):7213–7. 10.1073/pnas.86.18.7213 2550935 PMC298027

[28] Montefiori DC, Baba TW, Li A, Bilska M, Ruprecht RM. Neutralizing and infection-enhancing antibody responses do not correlate with the differential pathogenicity of SIVmac239delta3 in adult and infant rhesus monkeys. J Immunol. 1996;157(12):5528–35. 8955203

[29] Lambert AA, Gilbert C, Richard M, Beaulieu AD, Tremblay MJ. The C-type lectin surface receptor DCIR acts as a new attachment factor for HIV-1 in dendritic cells and contributes to trans- and cis-infection pathways. Blood. 2008;112(4):1299–307. 10.1182/blood-2008-01-136473 18541725 PMC2515113

[30] Chung AW, Rollman E, Center RJ, Kent SJ, Stratov I. Rapid degranulation of NK cells following activation by HIV-specific antibodies. J Immunol. 2009;182(2):1202–10. 10.4049/jimmunol.182.2.1202 19124764

[31] Kaul R, Plummer F, Clerici M, Bomsel M, Lopalco L, Broliden K. Mucosal IgA in exposed, uninfected subjects: evidence for a role in protection against HIV infection. AIDS. 2001;15(3):431–2. 10.1097/00002030-200102160-00026 11273233

[32] Hirbod T, Kaul R, Reichard C, Kimani J, Ngugi E, Bwayo JJ, et al. HIV-neutralizing immunoglobulin A and HIV-specific proliferation are independently associated with reduced HIV acquisition in Kenyan sex workers. AIDS. 2008;22(6):727–35. 10.1097/QAD.0b013e3282f56b64 18356602

[33] Lamm ME, Nedrud JG, Kaetzel CS, Mazanec MB. IgA and mucosal defense. APMIS. 1995;103(4):241–6. 10.1111/j.1699-0463.1995.tb01101.x 7612253

[34] Haynes BF, Gilbert PB, McElrath MJ, Zolla-Pazner S, Tomaras GD, Alam SM, et al. Immune-correlates analysis of an HIV-1 vaccine efficacy trial. N Engl J Med. 2012;366(14):1275–86. 10.1056/NEJMoa1113425 22475592 PMC3371689

[35] Ruiz MJ, Ghiglione Y, Falivene J, Laufer N, Holgado MP, Socias ME, et al. Env-Specific IgA from Viremic HIV-Infected Subjects Compromises Antibody-Dependent Cellular Cytotoxicity. J Virol. 2016;90(2):670–81. 10.1128/JVI.02363-15 26491172 PMC4702681

[36] Belec L, Georges AJ, Steenman G, Martin PM. Antibodies to human immunodeficiency virus in vaginal secretions of heterosexual women. J Infect Dis. 1989;160(3):385–91. 10.1093/infdis/160.3.385 2760496

[37] Dorrell L, Hessell AJ, Wang M, Whittle H, Sabally S, Rowland-Jones S, et al. Absence of specific mucosal antibody responses in HIV-exposed uninfected sex workers from the Gambia. AIDS. 2000;14(9):1117–22. 10.1097/00002030-200006160-00008 10894275

[38] Buchacz K, Parekh BS, Padian NS, van der Straten A, Phillips S, Jonte J, et al. HIV-specific IgG in cervicovaginal secretions of exposed HIV-uninfected female sexual partners of HIV-infected men. AIDS Res Hum Retroviruses. 2001;17(18):1689–93. 10.1089/08892220152741388 11788020

[39] Schmitz JE, Kuroda MJ, Santra S, Simon MA, Lifton MA, Lin W, et al. Effect of humoral immune responses on controlling viremia during primary infection of rhesus monkeys with simian immunodeficiency virus. J Virol. 2003;77(3):2165–73. 10.1128/jvi.77.3.2165-2173.2003 12525651 PMC140983

[40] Jaworski JP, Kobie J, Brower Z, Malherbe DC, Landucci G, Sutton WF, et al. Neutralizing polyclonal IgG present during acute infection prevents rapid disease onset in simian-human immunodeficiency virus SHIVSF162P3-infected infant rhesus macaques. J Virol. 2013;87(19):10447–59. 10.1128/JVI.00049-13 23885083 PMC3807376

[41] Evans DT, Knapp LA, Jing P, Mitchen JL, Dykhuizen M, Montefiori DC, et al. Rapid and slow progressors differ by a single MHC class I haplotype in a family of MHC-defined rhesus macaques infected with SIV. Immunol Lett. 1999;66(1–3):53–9. 10.1016/s0165-2478(98)00151-5 10203034

[42] Shedlock DJ, Silvestri G, Weiner DB. Monkeying around with HIV vaccines: using rhesus macaques to define ’gatekeepers’ for clinical trials. Nat Rev Immunol. 2009;9(10):717–28. 10.1038/nri2636 19859066 PMC4317297

[43] Guimaraes MD, Vlahov D, Castilho EA. Postcoital vaginal bleeding as a risk factor for transmission of the human immunodeficiency virus in a heterosexual partner study in Brazil. Rio de Janeiro Heterosexual Study Group. Arch Intern Med. 1997;157(12):1362–8. 10.1001/archinte.1997.00440330102012 9201011

[44] Weir SS, Roddy RE, Zekeng L, Feldblum PJ. Nonoxynol-9 use, genital ulcers, and HIV infection in a cohort of sex workers. Genitourin Med. 1995;71(2):78–81. 10.1136/sti.71.2.78 7744418 PMC1195458

[45] Zhu J, Hladik F, Woodward A, Klock A, Peng T, Johnston C, et al. Persistence of HIV-1 receptor-positive cells after HSV-2 reactivation is a potential mechanism for increased HIV-1 acquisition. Nat Med. 2009;15(8):886–92. 10.1038/nm.2006 19648930 PMC2723183

[46] Pattacini L, Murnane PM, Baeten JM, Fluharty TR, Thomas KK, Bukusi E, et al. Antiretroviral Pre-Exposure Prophylaxis Does Not Enhance Immune Responses to HIV in Exposed but Uninfected Persons. J Infect Dis. 2015;211(12):1943–52. 10.1093/infdis/jiu815 25520426 PMC4836720

[47] Barnett SW, Srivastava IK, Kan E, Zhou F, Goodsell A, Cristillo AD, et al. Protection of macaques against vaginal SHIV challenge by systemic or mucosal and systemic vaccinations with HIV-envelope. AIDS. 2008;22(3):339–48. 10.1097/QAD.0b013e3282f3ca57 18195560

[48] Tsegaye TS, Butler K, Luo W, Radzio J, Srinivasan P, Sharma S, et al. Repeated Vaginal SHIV Challenges in Macaques Receiving Oral or Topical Preexposure Prophylaxis Induce Virus-Specific T-Cell Responses. J Acquir Immune Defic Syndr. 2015;69(4):385–94. 10.1097/QAI.0000000000000642 25886925 PMC4485592

[49] Hamajima K, Hoshino Y, Xin KQ, Hayashi F, Tadokoro K, Okuda K. Systemic and mucosal immune responses in mice after rectal and vaginal immunization with HIV-DNA vaccine. Clin Immunol. 2002;102(1):12–8. 10.1006/clim.2001.5141 11781062

[50] Wang SW, Bertley FM, Kozlowski PA, Herrmann L, Manson K, Mazzara G, et al. An SHIV DNA/MVA rectal vaccination in macaques provides systemic and mucosal virus-specific responses and protection against AIDS. AIDS Res Hum Retroviruses. 2004;20(8):846–59. 10.1089/0889222041725253 15320989

[51] Durrani Z, McInerney TL, McLain L, Jones T, Bellaby T, Brennan FR, et al. Intranasal immunization with a plant virus expressing a peptide from HIV-1 gp41 stimulates better mucosal and systemic HIV-1-specific IgA and IgG than oral immunization. J Immunol Methods. 1998;220(1–2):93–103. 10.1016/s0022-1759(98)00145-8 9839930

[52] Brave A, Hallengard D, Schroder U, Blomberg P, Wahren B, Hinkula J. Intranasal immunization of young mice with a multigene HIV-1 vaccine in combination with the N3 adjuvant induces mucosal and systemic immune responses. Vaccine. 2008;26(40):5075–8. 10.1016/j.vaccine.2008.03.066 18450334

[53] Pun PB, Bhat AA, Mohan T, Kulkarni S, Paranjape R, Rao DN. Intranasal administration of peptide antigens of HIV with mucosal adjuvant CpG ODN coentrapped in microparticles enhances the mucosal and systemic immune responses. Int Immunopharmacol. 2009;9(4):468–77. 10.1016/j.intimp.2009.01.012 19291836

[54] Tomusange K, Wijesundara D, Gummow J, Wesselingh S, Suhrbier A, Gowans EJ, et al. Mucosal vaccination with a live recombinant rhinovirus followed by intradermal DNA administration elicits potent and protective HIV-specific immune responses. Sci Rep. 2016;6:36658. 10.1038/srep36658 27853256 PMC5113119

